# 

**Published:** 1918-11

**Authors:** 


### ANNOUNCEMENTS

The Fifty-Third Annual Meeting of the Ohio State Dental Society will be held in Memorial Hall, Columbus, December 3, 4, and 5, 1918. An excellent program of papers and clinics is assured with some new features of especial interest.

Members in good standing of other state societies are cordially invited to meet with us.—F. R. CHAPMAN, *Sec.*, 305 Schultz Bldg., Columbus, Ohio.

---

### PREPAREDNESS LEAGUE OF AMERICAN DENTISTS
### NOTES AND NEWS

*Communication from the President*—Our Director-General is planning to give, through the members of the League, free dental service to such families of soldiers, sailors and marines as shall be found worthy by the Home Service Department of the Red Cross.

This is a most commendable activity of the League and should be supported by its entire membership. Only necessary service will be rendered, with special reference to children and expectant mothers who will, doubtless, make up the greater percentage of cases requiring treatment.

It is possible that some members of the League may not be in full accord with this plan, but to such I would say that we have thus far undertaken no more necessary nor worthy activity. Suppose that you were in the trenches more than three thousand miles away from your wife and your children who were left without sufficient support, suffering far more than yourself, in patience and silence. Would you not be cheered and thankful to know that skilled dentists stand ready to give them the best service within their power? Would it not fill your heart with thanksgiving and make your cross easier to bear?

Then, for the sake of your conscience, your love of your own flesh and blood, do not advance a single argument against this great work, but add it to your already overburdened duties and the blessings of real sacrifice will be yours throughout your lifetime. It is far better to sacrifice than to regret. Now is the time when every ounce of skilled energy must be utilized to the glory of our flag, our profession and humanity. All true Americans are learning to give and to sacrifice and surely no more worthy objects can be found than the wives and children of the men who are making the supreme sacrifice that our beloved country may remain clean and fit for the living as well as for the myriads yet to be born.

---

*The Red Cross and the Dental Motor Car*—The Red Cross will accept and ship to France all dental motor cars turned over to the Government by the League, and promises that they will be used for care of the soldiers only. With this assurance the League is ready to begin active work in supplying them as rapidly as possible.

Members of the Dental Corps in France are anxiously awaiting their arrival and we have many letters urging us to get the cars over there as there is the greatest need for them. Commanding officers have promised co-operation and it is now up to the League to supply the demand.

Fifteen cars have been completed, some of which were ordered by the Italian Government for child welfare service. No development has created greater interest in our profession, especially by the public, than our dental motor car.

Let every organized body of dentists plan immediately to raise money for this purpose and I suggest that the officers write Dr. S. Marshall Weaver, *Chairman*, 620 Rose Bldg., Cleveland, Ohio, for full instructions.

*This matter is vital, therefore urgent.*

---

*Our Instruction Course*—Conditions generally in the educational department of our profession have been so unsettled while undergoing the changes subsequent upon militarizing dental institutions that those in charge of arranging a course of instruction in war oral and dental surgery have been unable to make definite arrangements in this most essential matter.

In the meantime, data is being compiled, plans formulated and co-operation of instructors gained so that when the word is given to proceed we will be ready for action. The situation has worried your President greatly as he has endeavored to establish a reputation for the League of accomplishing every good object undertaken and it is no idle assurance to say that we are on the job to see this object a big success.

*Reclaim*—Colonel Logan asks all League members to reclaim men dentally defective and put them in Class 1 A. We will back him up in this and do our bit to make an army of five millions. *The war is not yet won.*—J. W. Beach, *President.*

---

### AMERICAN RED CROSS DENTAL AMBULANCES

And now they are utilizing dental ambulances in France, that American soldiers may have healthy mouths, with consequent good digestion and health. The first two of these dental ambulances on record were equipped through the efforts of Mrs. William Boyce Thompson and given through the American Red Cross to the Dental Department of the United States Army.

These ambulances have all the appurtenances of the modern dental office, with the added advantage of being able to visit the patient in all sorts of out-of-the-way places. They consist of a main operating room, and two good-sized tents. Every contrivance approved by the dental profession is installed in the ambulance.

In the center room, or office proper, is a complete dental outfit, with separate bracket for instruments, electric engine, fountain cuspidor, pressure tank with syringe and sprays, hot and cold water faucets, steam sterilizer, filter, vulcanizer, electric lathe, blow pipe, nitrous oxide and oxygen gas apparatus, sanitary cabinet for instruments, a full set of forceps and duplicate sets of instruments. There is even a typewriter for office record use.

The car is lighted by electricity generated by batteries especially built for the ambulance. Should they fail, provision is made for acetylene gas lighting.

When the car is in transit a field outfit, which closes up in three containers, is stored in the office. This is set up as soon as the car is ready for service. On either side of the office is a tent, one used for the field outfit; the other is converted into sleeping quarters or it may be used as an adjunct operating room.

While dental treatment is being given in the office, one of the tents may be used for the more difficult operations. The car is manned by a crew of two officers, two dental assistants and a chauffeur. In hot weather the sides of the tent may be rolled up, insuring all possible ventilation.

One of these ambulances is in active service at Camp Upton, the other at Camp Greenleaf. In all camps, newly arrived draftees carry with them trifling ailments—

measles, mumps, and the like. The men are quarantined and as they can not go to the dentist, the dental office honks up to them.

Camp Meade also has a dental ambulance, the gift of the Cleveland Unit of the Preparedness League. The Connecticut Unit provided funds for another which is going to France to serve our boys at the front. The Red Cross has thirteen of the dental ambulances, either completed or in process of construction. Three of them are for service in France and the other ten (three dental ambulances and seven baby saving trucks) are on their way to Italy. All thirteen will go ''over there.'' The Red Cross considers that the work among the babies and children of the war-swept lands will do much to rehabilitate future generations.

In order to continue this work and to provide for the greatly increased number of men who are being sent overseas monthly, the Red Cross will need the united support of the American people, that its mission of mercy and humanity may not be halted. For that reason the Red Cross will hold its second annual Christmas roll call during the week of December 16th to 23d, when, it is hoped, every American man, woman and child will place his or her name on the Red Cross roster. Last year 22,000,000 adults and 8,000,000 children responded to the call and took a nation-wide pledge to stand squarely behind the Flag in our war for justice and liberty for all.

The above paragraphs, under the heading, ''American Red Cross Dental Ambulances'' is matter which has been received direct from the Publicity Bureau of the Eastern Department of the Red Cross. It is therefore authentic. All League officers are requested to have this published in their local newspapers during the next Red Cross Drive, which will begin December 16th.—R. Ottolengui, Director of Publicity.

